# Synthesis, Electrochemical and Photochemical Properties of Sulfanyl Porphyrazine with Ferrocenyl Substituents

**DOI:** 10.3390/molecules28135215

**Published:** 2023-07-05

**Authors:** Mina Hassani, Amanda Leda, Weronika Porolnik, Michal Falkowski, Tomasz Rębiś, Jaroslaw Piskorz, Lukasz Popenda, Michal Wicinski, Dariusz T. Mlynarczyk, Nejat Düzgüneş, Michal P. Marszall

**Affiliations:** 1 Department of Medicinal Chemistry, Collegium Medicum in Bydgoszcz, Faculty of Pharmacy, Nicolaus Copernicus University in Torun, A. Jurasza 2, 85-089 Bydgoszcz, Poland; 503341@doktorant.umk.pl (M.H.); mmars@cm.umk.pl (M.P.M.); 2 Institute of Chemistry and Technical Electrochemistry, Poznan University of Technology, Berdychowo 4, 60-965 Poznan, Poland; amanda.leda@doctorate.put.poznan.pl; 3 Chair and Department of Inorganic and Analytical Chemistry, Poznan University of Medical Sciences, Rokietnicka 3, 60-806 Poznan, Poland; w.porolnik@op.pl (W.P.); piskorzj@ump.edu.pl (J.P.); 4 NanoBioMedical Centre, Adam Mickiewicz University, Wszechnicy Piastowskiej 3, 61-614 Poznan, Poland; lpopenda@amu.edu.pl; 5 Department of Pharmacology and Therapy, Collegium Medicum in Bydgoszcz, Faculty of Medicine, Nicolaus Copernicus University in Torun, Curie Sklodowskiej 9, 85-094 Bydgoszcz, Poland; michal.wicinski@cm.umk.pl; 6 Chair and Department of Chemical Technology of Drugs, Poznan University of Medical Sciences, Grunwaldzka 6, 60-780 Poznan, Poland; mlynarczykd@ump.edu.pl; 7 Department of Biomedical Sciences, Arthur A. Dugoni School of Dentistry, University of the Pacific, San Francisco, CA 94103, USA; nduzgunes@pacific.edu

**Keywords:** ferrocene, singlet oxygen, porphyrazine, voltammetry, macrocyclic compound

## Abstract

Ferrocene is useful in modern organometallic chemistry due to its versatile applications in material sciences, catalysis, medicinal chemistry, and diagnostic applications. The ferrocene moiety can potentially serve many purposes in therapeutics and diagnostics. In the course of this study, (6-bromo-1-oxohexyl)ferrocene was combined with dimercaptomaleonitrile sodium salt to yield a novel maleonitrile derivative. Subsequently, this compound was subjected to an autocyclotetramerization reaction using the Linstead conditions in order to obtain an octaferrocenyl-substituted magnesium(II) sulfanyl porphyrazine. Following that, both compounds—the maleonitrile derivative and the porphyrazine derivative—were subjected to physicochemical characterization using UV-Vis, ES-TOF, MALDI-TOF, and one-dimensional and two-dimensional NMR spectroscopy. Moreover, the sulfanyl porphyrazine was subjected to various photophysical studies, including optical absorption and emission measurements, as well as the evaluation of its photochemical properties. Values of singlet oxygen generation quantum yields were obtained in different organic solvents. The electrochemical properties of the synthesized compounds were studied using cyclic voltammetry. According to the electrochemical results, the presence of electron-withdrawing oxohexyl groups attached to ferrocene afforded significantly more positive oxidation potentials of the ferrocene-based redox process up to 0.34 V vs. Fc^+^/Fc.

## 1. Introduction

Ferrocene is a metalloorganic compound that constantly catches the attention of chemists, materials researchers, and pharmacists. Although it was not the first organometallic complex reported, its discovery is considered to be the spark that started the differentiation of organometallic chemistry as a separate area of chemistry [1]. The ferrocene moiety can potentially serve many purposes in therapeutics and diagnostics, as it was found to be a sensitive probe, a chromophore, a biological marker, a redox-active moiety, and a catalytic fragment, among others [2]. Ferrocene is also known to express biological activity as a scaffold or a fragment of the chromophore, with significant anticancer, antimicrobial, and antimalarial activity [3]. Other biological activities associated with compounds possessing the ferrocene motif in their structures include antipyretic, anti-inflammatory, analgesic, and anti-hyperglycemic activity [2].

Porphyrazines (Pzs) are synthetic tetrapyrrolic macrocycles, which are aza analogs of naturally occurring porphyrins. The highly conjugated porphyrazine ring grants the molecule unique optical, electrical, and photochemical properties, rendering it potentially useful in fields such as medicine, sensors, and photocatalysis [4,5,6]. The unsubstituted porphyrazine ring is a highly nonpolar molecule that is prone to the phenomenon of π–π stacking, significantly hampering its broad use. Thus, to improve its properties porphyrazines have to be functionalized in two main approaches: by introducing or changing the ion in the macrocyclic core’s coordinating center or by the introduction of peripheral substituents at the pyrrole β-positions. Owing to such modification, the physicochemical properties of the porphyrazine may be tailored to specific applications. Among Pzs, sulfanyl Pzs have gained attention, as they were found to express good solubility and show biological activity [7,8], and to present interesting photocatalytic [9,10], electronic [11,12], and optical properties [13]. The sulfanyl Pzs bearing nitrophenoxy [14,15] and isophthaloxyalkyl substituents [16,17] recently synthesized in our group revealed a plethora of interesting photochemical and electrochemical properties. Porphyrinoids, including aza-porphyrinoids, have already been the subject of studies aiming to combine them with ferrocene [18,19]. Such combinations were found to mimic photosynthesis-active sites and were promising composites for electronic devices and molecular electrochemical sensors. Depending on the mode of connection between the two moieties, the resulting dyads may present properties either showing the separate combination of the porphyrinoid and ferrocene, or resultant properties of the combination where both molecules affect each other. The possibility of attaching several ferrocenyl moieties to the porphyrinoid has a great impact on its electronic properties and makes such molecules suitable candidates for applications as photovoltaic light harvesting and energy conversion systems, as well as intelligent surface coatings [20].

In this work, we report the synthesis and properties of novel maleonitrile and sulfanyl porphyrazine derivatives with peripheral ferrocenyl moieties. Both compounds were characterized using spectral methods including mass spectrometry, NMR spectroscopy, and UV-Vis spectrophotometry. Furthermore, the photochemical properties were assessed by means of the quantum yield of singlet oxygen generation. The voltammetric data clearly indicated defined redox features corresponding to one-electron reactions of the π-conjugated porphyrazine ring and ferrocene substituents in the periphery.

## 2. Results and Discussion

### 2.1. Synthesis and Characterization

**Scheme 1:**
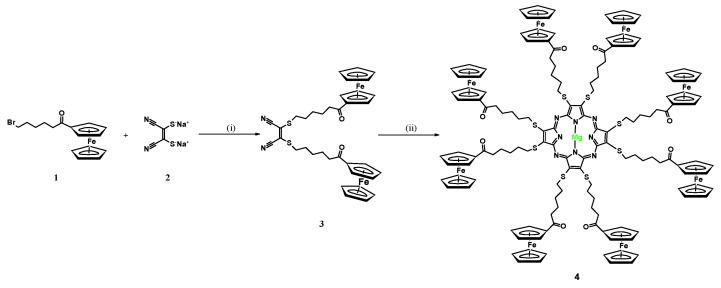
Synthesis of compounds **3** and **4**. Reagents and conditions: (i) K_2_CO_3_, DMF, 50 °C, 72 h; (ii) Mg(OC_4_H_9_)_2_, *n*-butanol, reflux, 20 h.

A new porphyrazine derivative was prepared using a two-step synthetic pathway. First, the alkylation reaction of (6-bromo-1-oxohexyl)ferrocene (**1**) with dimercaptomaleonitrile disodium salt (**2**) in *N*,*N*-dimethylformamide with potassium carbonate as a base, following methodology developed before [21], led to novel maleonitrile derivative **3**. Subsequently, after its isolation from the reaction mixture using chromatographical methods, compound **3** was used in a Linstead tetramerization reaction in *n*-butanol with the use of magnesium *n*-butoxide as a base to form a novel symmetrical magnesium(II) porphyrazine **4** (Scheme 1). As opposed to the convergent approach utilized in this study, a divergent synthetic approach to similar octakis(ferrocene)-substituted porphyrazine derivatives was proposed by Akkus and Gül [22]. In the initial step, they synthesized the magnesium(II) porphyrazine core with hydroxyl peripheral groups, to which ferrocene moieties were attached afterward by means of an esterification reaction.

The Pz **4** was subjected to purity assessment, and HPLC analyses performed in three different solvent systems confirmed the purity of the new macrocycle using detection at 370 and 670 nm (see Supplementary Data). All obtained compounds were purified using flash column chromatography and characterized by mass spectrometry and UV-Vis spectroscopy. To unambiguously identify the isolated macrocycle, NMR experiments were carried out.

**Figure 1:**
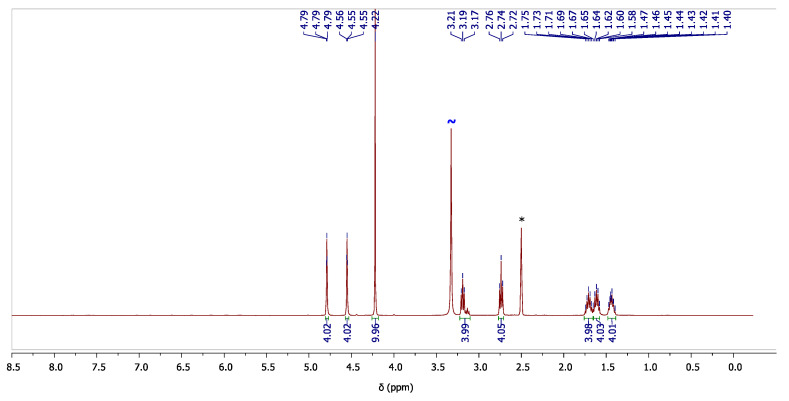
^1^H NMR spectrum of **3** (DMSO-*d*_6_, 298 K). The symbols * and ~ indicate DMSO-*d*_6_ and water residual peaks, respectively.

**Figure 2:**
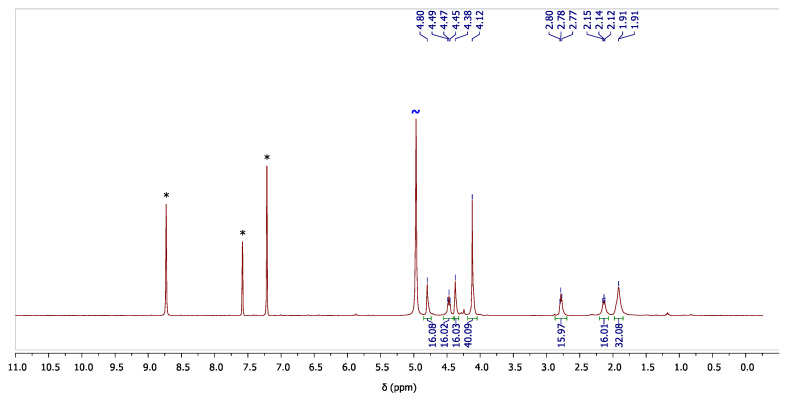
^1^H NMR spectrum of **4** (pyridine-*d*_5_, 298 K). The symbols * and ~ indicate pyridine-*d*_5_ and water residual peaks, respectively.

In the ^1^H NMR spectrum of the maleonitrile derivative **3** (Figure 1), the ferrocenyl moiety CH groups appear as singlets at 4.22 ppm in the unsubstituted ring, while the CH groups in the substituted ring were observed as triplets at 4.55 and 4.79 ppm, for the hydrogens in the α and β positions, respectively. Signals of the aliphatic chain protons were assigned on the basis of ^1^H-^1^H COSY and ^1^H-^13^C HMBC experiments, from the nearest to the sulfur atom: 3.19 (triplet), 1.71 (multiplet), 1.44 (multiplet), 1.62 (multiplet), and 2.74 (triplet). The corresponding carbon atoms were assigned based on the correlations in the ^1^H-^13^C HSQC spectrum. The quaternary carbons were identified using the ^1^H-^13^C HMBC spectrum: the 79.0 ppm peak—corresponded to the ferrocene carbon in the 1 position; 112.3 ppm—carbon of the cyano group; 120.9 ppm—sp^2^ carbon in the maleonitrile fragment; and the peak at 203.1 ppm corresponded to the carbonyl group carbon (Figure 2).

The ^1^H NMR spectrum of the porphyrazine **4** shares several similarities with the spectrum recorded for **3**. Again, the signals of unsubstituted ferrocene ring CH groups appeared upfield at 4.12 ppm as a triplet. The CH groups at the α and β positions of the substituted cyclopentadienyl ring were observed at 4.38 and 4.80 ppm, respectively. In this case, however, they were not observed as triplets but as broad lines. The change in the chemical shift of the aliphatic linker protons was more pronounced the closer they were to the macrocyclic ring. The proton signal for CH_2_ group neighboring the carbonyl (furthest from the macrocycle) was observed at 2.78 ppm, and for SCH_2_, it was observed at 4.47 ppm. To compare, the signals of the corresponding groups in **3** were detected at 2.74 ppm and 3.19 ppm, respectively. The carbon chemical shifts in the corresponding groups were also recorded slightly downfield, but there was no relation in regard to the distance to the macrocyclic ring. As expected, the ^13^C NMR signals recorded for the maleonitrile fragment in **3** were not present. Instead, two new signals were observed at 158.4 and 141.8 ppm, corresponding to the α and β positions of the pyrrole ring (Figures S1–S4).

To unambiguously determine the chemical structure of the prepared compounds, we attempted to obtain single crystals to perform single-crystal X-ray diffraction; however, up until the submission of this manuscript, no success in this matter was achieved.

### 2.2. Optical Properties

**Figure 3:**
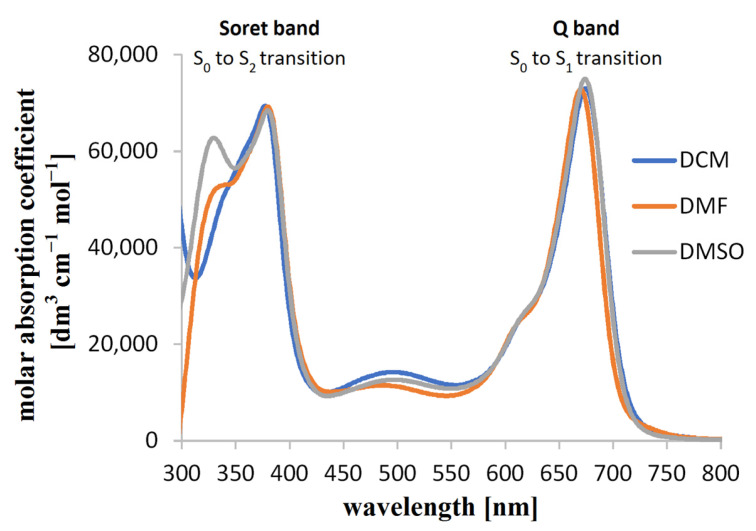
UV-Vis absorption spectra of Pz **4** in dichloromethane (DCM), *N*,*N*-dimethylformamide (DMF), and dimethylsulfoxide (DMSO).

The UV-Vis spectra of porphyrazine **4**, recorded in the organic solvents *N,N*-dimethylformamide, dimethylsulfoxide, and dichloromethane, revealed absorption properties typical for porphyrazine macrocycles (Figure 3). The short-wavelength Soret band exhibited absorption maxima at 380 nm in *N,N*-dimethylformamide and dimethylsulfoxide, and at 378 nm in dichloromethane. Long-wavelength Q bands with maxima in the 671–675 nm range were also observed. The Soret band results from the transition between the ground state and the second singlet excited state (S_0_ → S_2_), whereas the Q band corresponds to the transition from the ground state to the first singlet excited state (S_0_ → S_1_) [23,24].

The calculated values of the logarithms of the molar absorption coefficients (log ε) for these bands were in the range of 4.84–4.88 (Table 1). In addition, a low-intensity broad band was observed at about 425–525 nm and can be assigned to *n*-sulfur → π * transitions [13,25]. These optical properties were similar to those of other sulfanyl porphyrazines, indicating that peripheral ferrocene substituents did not significantly influence absorption features [26,27,28].

The fluorescence studies performed for the DMF and DMSO solutions of Pz **4** did not reveal any emission properties. The literature data indicated that emission quenching by sulfur atoms combined with the porphyrazine core could be a reason for the lack of fluorescence [29,30].

### 2.3. Photosensitized Production of Singlet Oxygen

**Figure 4:**
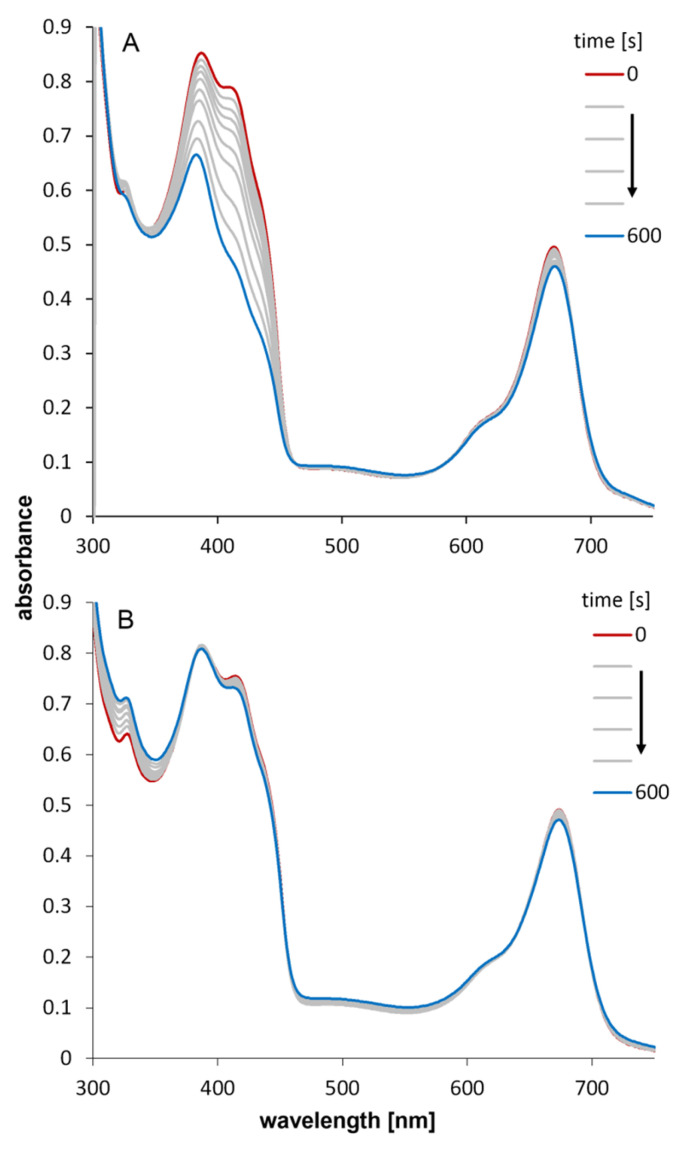
Changes in the UV-Vis spectra during irradiation of Pz **4** and DPBF in (**A**) *N*,*N*-dimethylformamide and (**B**) dimethylsulfoxide.

Porphyrazine **4** was also evaluated for potential photosensitizing activity by measuring the quantum yield of singlet oxygen generation (Φ_Δ_). The mechanism of singlet oxygen generation is connected with the type II photodynamic reaction. Upon light absorption, the photosensitizer molecule is transformed from the ground state (S_0_) to the singlet excited state (S_1_) and then to the triplet state (T_1_) via the intersystem crossing process. Next, the energy can be transferred directly to the molecular oxygen in the ground triplet state, resulting in singlet oxygen formation [31]. Singlet oxygen is one of the main reactive oxygen species responsible for the cytotoxic effect of photodynamic therapy [32,33]. The comparative method was utilized for Φ_Δ_ measurements with zinc(II) phthalocyanine as a reference and 1,3-diphenylisobenzofuran (DPBF) as a chemical quencher of singlet oxygen (Figure 4) [34,35]. Pz **4** revealed a low ability for singlet oxygen generation, with Φ_Δ_ values of 0.064 and 0.004 in DMF and DMSO, respectively (Table 1). We also observed changes in the Pz **4** absorption, which indicates that the minor photodegradation process of Pz **4** also occurred during the singlet oxygen generation measurement. After 10 min of irradiation, the degradation of Pz **4** equaled 7.5% in DMF and 4.3% in DMSO solution.

A low tendency of Pz **4** to generate singlet oxygen with a simultaneous lack of emission properties suggests the existence of other deactivation processes of the excited state. Further advanced studies, including photoacoustic imaging and spectroscopy, are needed to determine possible non-radiative deactivation processes, including the vibration relaxation of chromophore molecules [36,37].

### 2.4. Electrochemical Characterization of Studied Compounds in TBAP/DCM Electrolyte

**Figure 5:**
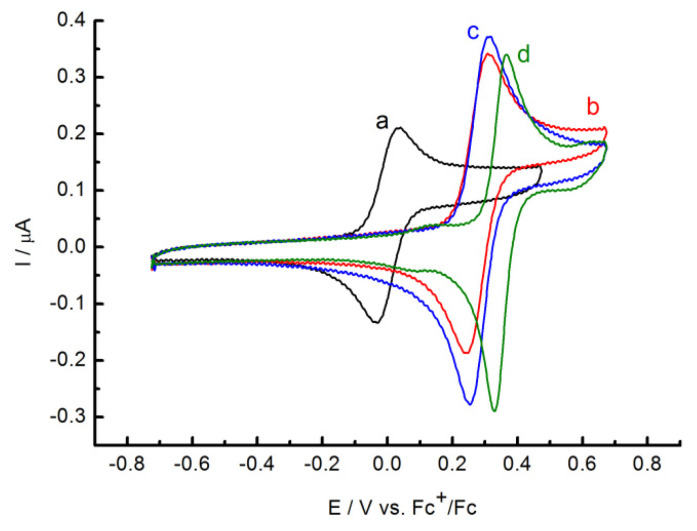
Cyclic voltammogram for Fc (a) **1** (b), **3** (c), and **4** (d) recorded in 0.05 M DCM/TBAP at a scan rate of 50 mV s^−1^.

The electrochemical properties of the synthesized compounds were studied using cyclic voltammetry (CV) in 0.05 M TBAP/DCM electrolyte, as shown in Figure 5. Control measurements of 0.1 mM ferrocene (Fc) were also performed for comparative purposes. The observed major peaks of the compounds are ascribed to the one-electron transfer of the substituted ferrocene groups. The obtained data show that the ferrocene-based redox processes of **1**, **3**, and **4** were shifted towards more positive potentials in comparison to unsubstituted Fc. The formal potentials of **1** and **3** were equal to E^0^′ = 0.28 V vs. Fc^+^/Fc, and the E^0^′ for **4** was equal to 0.34 V vs. Fc^+^/Fc. This positive shift can be assigned to the electron-withdrawing effect of the oxohexyl groups, thus disfavoring the electron removal from the reduced form of the ferrocene group by the electrode and favoring the electron uptake by the oxidized form of ferrocene from the electrode [38,39,40]. The most positive peak potential observed for **4** (0.34 V) was probably correlated to the strongest inductive effect of the eight substituents existing close together in the **4** macrocycle. It is well known from previous publications that the redox potential of ferrocene can be tuned by the substitution of various groups. For instance, Paul et al. [38] presented a series of ferrocene-substituted electro-donating groups, such as methyl and *tert*-butyl. The substituents made it possible to shift the potential of the redox pair, even by about 0.5 V, towards a more negative potential than Fc [41]. In another work, Manfredi et al. presented a group of ferrocene derivatives with electron-withdrawing groups, such as thiophene rings bearing either alkyl or benzene derivatives bearing electron-withdrawing groups [42]. They observed a positive shift of the formal potential up to 0.35 V vs. Fc. Moreover, Battrejee et al. presented fifteen ferrocene-based compounds with substituents that strongly affect the redox peak position of Fc^+^/Fc. The authors observed a positive potential shift for p-methyl benzalacetylferrocene and ferrocylidene acetophenones. In the case of **1**, where only one oxohexyl group was anchored to the ferrocene, we observed a 0.28 V shift towards more positive potentials, exhibiting that oxohexyl groups possess moderate electron-withdrawing properties.

**Figure 6:**
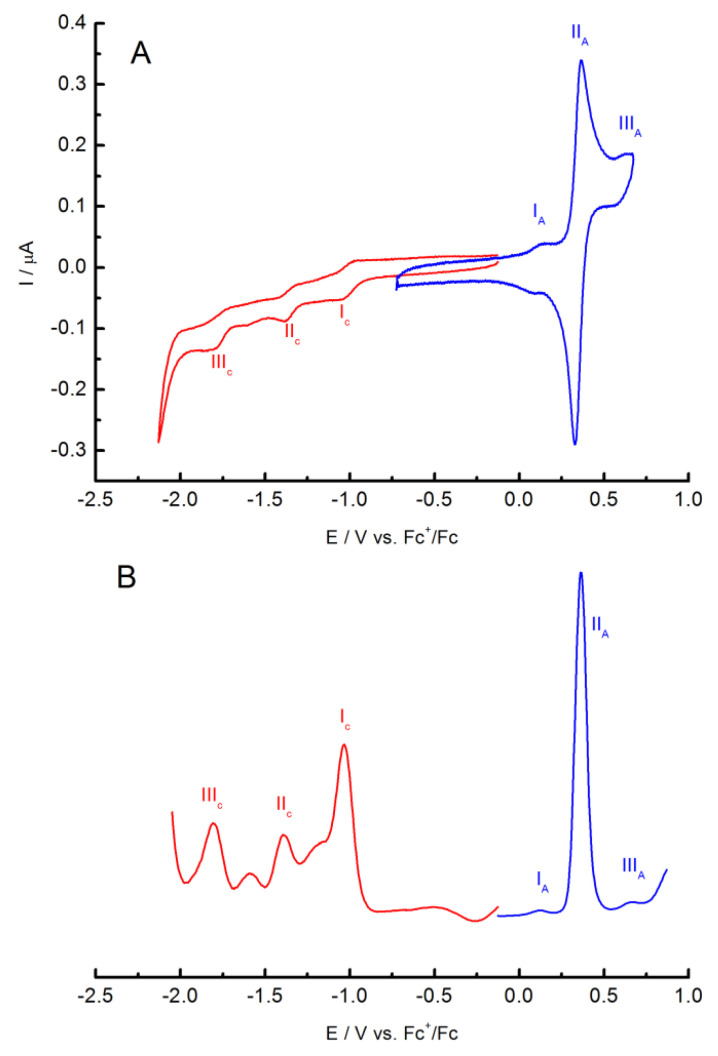
Cyclic voltammograms of **4** recorded in 0.05 M TBAP/DCM in a broad potential range at a scan rate of 50 mV s^−1^ (**A**). Differential pulse voltammograms for **4** in the same electrolyte (**B**).

Apart from ferrocene-based redox peaks (labeled as II_A_), **4** exhibited additional features due to the electroactivity of the macrocyclic core [21,43,44]. It can be seen in Figure 6A,B that two additional peaks at 0.13 (labeled as I_A_) and 0.60 V (labeled as III_A_) are visible. These peaks originated from one-electron redox processes occurring at the electron-rich macrocycle. Moreover, in the cathodic range, three well-defined redox features can be distinguished at −1.0 V (labeled as I_C_), −1.3 V (labeled as II_C_), and −1.80 V (labeled as III_C_). Such processes can also be assigned to the one-electron transfer within the macrocycle.

**Figure 7:**
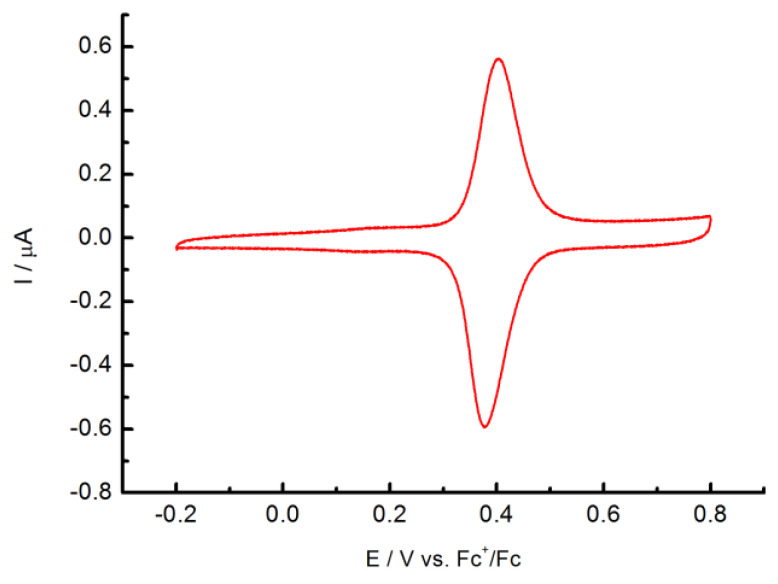
CV recorded in blank TBAP/DCM for GC electrode that was previously immersed in a solution of **4**. The scan rate was 100 mV s^−1^.

Porphyrazine **4** tended to adsorb on the electrode surface. Figure 7 presents CV data recorded in blank TBAP/DCM for a glassy carbon (GC) electrode that was previously immersed in a solution of **4**. Well-defined redox coupling occurred at E^0^′ = 0.39 V. The peak-to-peak separation was only 20 mV, suggesting a surface-confined process. Such behavior could be due to the electron-rich, hydrophobic nature of the studied compounds that enables adsorption of the **4** to the surface via π-π interactions.

**Figure 8:**
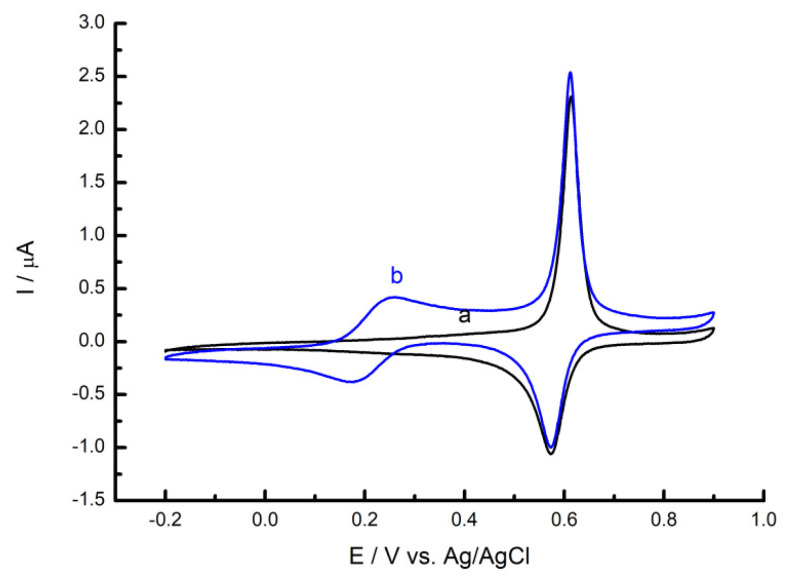
Cyclic voltammograms for a GC/MWCNT/**4** electrode recorded in 0.1 M HClO_4_ (curve a) and in 0.1 M HClO_4_ containing 0.5 mM Fc (curve b).

To obtain more insight into the adsorptive properties of **4**, we deposited **4** on the GC electrode covered by multi-walled carbon nanotubes (MWCNT). Various macrocyclic compounds have been previously deposited on the surface of MWCNT in order to construct electrocatalytic sensors [45,46,47,48]. The resultant GC/MWCNT/**4** was investigated using CV in an aqueous 0.1 M HClO_4_ electrolyte (Figure 8). The GC/MWCNT/**4** exhibited well-defined and sharp peaks that shifted positively at ca. 0.35 V versus ferrocene (curve b). The asymmetry of anodic-to-cathodic peak currents were visible. The anodic peak was ca. 1.7 times higher than the cathodic peak, which suggests some limitations in electron transfer between Fc^+^/Fc couples and MWCNT. The hampered electron transfer kinetics could be the result of the bulky structure of **4.**

## 3. Materials and Methods

### 3.1. General Procedures

All reactions were conducted in oven-dried glassware under an argon atmosphere. All solvents were rotary-evaporated at or below 60 °C under reduced pressure. The reported reaction temperatures refer to Radleys^®^ Heat-On display temperatures. Dry flash column chromatography was carried out on Merck silica gel 60 (Merck, Darmstadt, Germany) with a particle size of 40–63 μm and reverse phase on a Fluka C18 silica gel 90 (Merck, Darmstadt, Germany). Thin-layer chromatography (TLC) was performed on silica gel Merck Kieselgel 60 F254 plates (Merck, Darmstadt, Germany) and visualized with UV light (λ_max_ = 254 or 365 nm). NMR spectra were recorded at 298 K on a Bruker Avance III 500 spectrometer operating at resonance frequencies of 500.250 and 125.788 MHz for ^1^H and ^13^C, respectively. Chemical shifts (δ) are quoted in parts per million (ppm) and are referenced to residual solvent peaks: 3.330 and 2.500 ppm DMSO-*d*_6_ for ^1^H NMR and 39.520 ppm for ^13^C NMR, and 8.740, 7.580, and 7.220 ppm pyridine-*d*_5_ for ^1^H NMR and 150.350, 135.910, and 123.870 ppm for ^13^C NMR. Coupling constants (*J*) are quoted in Hertz (Hz). The abbreviations s, t, br, and m refer to singlet, triplet, broad signal, and multiplet, respectively. ^1^H and ^13^C resonances were unambiguously assigned based on ^1^H, ^13^C, ^1^H–^1^H COSY, ^1^H–^13^C HSQC, and ^1^H–^13^C HMBC spectra. Mass spectra (MALDI-TOF, HRMS) were carried out by the Wielkopolska Center for Advanced Technologies at the Adam Mickiewicz University in Poznan and at the European Center for Bioinformatics and Genomics in Poznan. UV-Vis spectra were recorded on a Shimadzu UV-1900i spectrophotometer (Duisburg, Germany). Dichloromethane (DCM, puriss. p.a., ≥99.9%) and tetrabutylammonium perchlorate (TBAP, ≥99.9%) were purchased from Merck (Darmstadt, Germany). Ferrocene (Fc) was provided by Alfa Aesar (Haverhill, MA, USA). All listed chemicals were reagent grade; thus, they did not require further purification. Dimercaptomaleonitrile disodium salt hydrate and (6-bromo-1-oxohexyl)ferrocene were purchased from Sigma-Aldrich (Merck, Darmstadt, Germany).

### 3.2. Synthetic Procedures and Characterization

(2Z)-2,3-bis{[(6-ferrocenyl-6-oxohexyl)]sulfanyl}but-2-enedinitrile (**3**) was synthesized by adapting a previously published procedure [28]. Dimercaptomaleonitrile disodium salt (200 mg, 1075 mmol) (**1**), (6-Bromo-1-oxohexyl)ferrocene (**2**) (975 mg, 2.69 mmol), and anhydrous K_2_CO_3_ (600 mg, 4.34 mmol) were dissolved in 20 mL of anhydrous *N*,*N*-dimethylformamide (DMF). The reaction mixture was stirred at 50 °C for 72 h. Next, the reaction mixture was filtered through celite, and the solid residue was washed with 200 mL of dichloromethane (DCM). The collected filtrates were evaporated under reduced pressure, and the residual brown oil was chromatographed using (DCM: methanol, 100: 1 to 20:1, *v/v*) to give **3** in the form of an orange-brown oil (501 mg, 66% yield). The R*f* (DCM:methanol, 50:1, *v/v*) was 0.29. UV-Vis (DCM) *λ*_max_ nm (log ε): 273 (4.82), 345 (4.31), (DMF) *λ*_max_ nm (log ε) 271 (4.36), and 347 (4.30). ^1^H NMR (500 MHz, DMSO-*d*_6_) δ, ppm: 4.79 (t, *J* = 1.8 Hz, 4H, C3-H, C4-H, FcH), 4.55 (t, *J* = 1.8 Hz, 4H, C2-H, C5-H, FcH), 4.22 (s, 10H, FcH), 3.19 (t, *J* = 7.3 Hz, 4H, SCH_2_), 2.74 (t, *J* = 7.1 Hz, 4H, COCH_2_), 1.71 (m, 4H, SCH_2_CH_2_), 1.62 (m, 4H, COCH_2_CH_2_), and 1.44 (m, 4H, SCH_2_CH_2_CH_2_). ^13^C NMR (125 MHz, DMSO-*d*_6_) δ, ppm: 203.1 (C=O), 120.9 (NC-C-S), 112.3 (CN), 79.0 (C1, ArC), 71.9 (C2, C5, FcC), 69.4 (FcC), 69.0 (C3, C4, FcC), 38.4 (COCH_2_), 34.2 (SCH_2_), 29.3 (SCH_2_CH_2_), 27.4 (SCH_2_CH_2_CH_2_), and 23.1 (COCH_2_CH_2_). HRMS (ESI) *m*/*z* found: 707.1178 [M + H]^+^, C_36_H_39_Fe_2_N_2_O_2_S_2_ requires 707.1152 [M + H]^+^; 729.0974 [M + Na]^+^, C_36_H_38_Fe_2_N_2_NaO_2_S_2_ requires 729.0971 [M+H]^+^.

2,3,7,8,12,13,17,18-Octakis[(6-ferrocenyl-6-oxohexyl)thio]porphyrazinato magnesium(II) (**4**) was synthesized using a modified literature procedure [28].

Magnesium turnings (15 mg, 0.621 mmol) and a small crystal of iodine were refluxed in *n*-butanol (10 mL) for 4 h. After cooling to room temperature, the reaction mixture was transferred to a flask containing **3** (0.439 mg, 0.621 mmol) and heated under reflux for another 20 h. Next, the reaction mixture was cooled to room temperature and filtered through Celite, which was subsequently washed with toluene (100 mL) and then DCM (100 mL). The combined filtrates were evaporated to dryness under reduced pressure, and a dark-blue residue was chromatographed using normal (DCM: methanol, 100:1 to 20:1, *v*/*v*), and reversed-phase systems (methanol, then DCM) to give **4** as a dark-blue film (0.143 mg, 32% yield). R*f* (DCM:methanol, 50:1, *v*/*v*) was 0.24. UV-Vis (DCM) λ_max_ nm (log ε): 378 (4.84), 675 (4.86). ^1^H NMR (500 MHz, pyridine-*d*_5_) δ, ppm: 4.80 (br, 16H, C3-H, C4-H, FcH), 4.47 (t, *J* = 7.0 Hz, 16H, SCH_2_), 4.38 (br, 16H, C2-H, C5-H, FcH), 4.12 (s, 40H, FcH), 2.78 (t, *J* = 6.1 Hz, 16H, COCH_2_), 2.14 (m, 16H, SCH_2_CH_2_), 1.92 (m, 16H, COCH_2_CH_2_), and 1.90 (m, 16H, SCH_2_CH_2_CH_2_). ^13^C NMR (125 MHz, pyridine-*d*_5_) δ, ppm: 203.8 (C=O), 158.4 (C1, C4, pyrrole-C), 141.8 (C2, C3, pyrrole-C), 80.4 (C1, ArC), 72.8 (C2, C5, FcC), 70.5 (FcC), 70.1 (C3, C4, FcC), 39.9 (COCH_2_), 35.9 (SCH_2_), 31.4 (SCH_2_CH_2_), 29.5 (SCH_2_CH_2_CH_2_), and 24.8 (COCH_2_CH_2_). MS (MALDI) *m*/*z*: found: 2849 [M + H]^+^ C_144_H_153_Fe_8_MgN_8_O_8_S_8_, requires 2849 [M + H]^+^. HPLC purity was 97.6–100.0% (see Supplementary Data).

### 3.3. Optical Properties

The electronic absorption UV-Vis spectra were recorded in the 200–900 nm range using a Shimadzu UV-1900i spectrophotometer and high-precision 1 cm light path quartz cells (Hellma Analytics, Jena, Germany). Molar absorption coefficients (ε) were calculated using the equation:

ε = A/(c × l)

where:

A—refers to the absorbance at the given wavelength; 

c—concentration of the solution [mol/dm^3^];

l—light path length.

A Jasco 6200 spectrofluorimeter was used to record emission spectra in DMF and DMSO solutions.

### 3.4. Photosensitized Production of Singlet Oxygen

The quantum yields of singlet oxygen generation (Φ_Δ_) were determined by the relative chemical trapping method [34,35,49]. 1,3-Diphenylisobenzofuran (DPBF, Aldrich) was used as a chemical quencher for singlet oxygen, and zinc(II) phthalocyanine was a reference with known singlet oxygen generation quantum yields (Φ_ΔDMF_ = 0.56; Φ_ΔDMSO_ = 0.67) [50]. Porphyrazine **4** and zinc(II) phthalocyanine were irradiated in DMF and DMSO solutions in the presence of DPBF with light of 670 nm from a 150 W high-pressure Xe lamp and a monochromator M250/1200/U (Optel, Opole, Poland). The light intensity was measured by a ThorLabs PM100D power and energy meter with a S120VC photodiode sensor that was set to 0.50 mW/cm^2^. The values of quantum yields were calculated using the equation:

$$\phi_{\Delta }=\phi_{\Delta }\frac{k_{\mathrm{sample}}}{k_{\mathrm{ref}.}}\frac{1-10^{-A_{\mathrm{ref}.}}}{1-10^{-A_{\mathrm{sample}}}}$$

where Φ_Δ_ refers to the singlet oxygen generation quantum yield and k is the constant rate of light-induced DPBF decomposition for the sample and the reference. The expression 1 − 10^−A^ is the absorption correction factor, with A referring to the absorbance at the irradiation wavelength for the reference and the sample.

### 3.5. Electrochemical Methods

An EmStat4S electrochemical analyzer was used for the electrochemical analysis (Eindhoven, The Netherlands). For non-aqueous measurements, a three-electrode system was set up, with a pseudo-reference electrode of a silver wire covered with AgCl and an auxiliary electrode of platinum wire. The working electrode was a glassy carbon electrode (GC) with a diameter of 1 mm (BASi, West Lafayette, IN, USA). Ferrocene (Fc) was introduced at the end of each test and the potential values were referred versus the Fc^+^/Fc redox couple. Before each electrochemical test, the GC electrode was polished with an aqueous suspension of alumina powder (Al_2_O_3_, with an average diameter of 50 nm, Buehler) on a polishing pad. Any impurities were subsequently removed using an ultrasonic bath (1:1 *v*/*v* solution of acetone and distilled water). Electrochemical measurements of **1**, **3**, and **4** were conducted in 0.05 M TBAP/DCM serving as the supporting electrolyte. For aqueous electrolyte measurements (0.1 M HClO_4_), Ag/AgCl (3 M KCl) served as the reference electrode. To prepare GC/MWCNT/**4,** 2 µL of MWCNT (Sigma-Aldrich, St. Louis, MO, USA) suspension in DMF (1 mg mL^−1^) was dropped on the surface of the GC. After drying in an oven (60 °C), the GC/MWCNT electrode was immersed in a solution of **4** (1 mg mL^−1^) for adsorption. The resultant GC/MWCNT/**4** was evaluated using CV.

## 4. Conclusions

The symmetrical magnesium porphyrazine derivative, possessing ferrocenyl substituents in the periphery, was synthesized and its physicochemical properties were analyzed using several methods, including UV spectrophotometry, NMR spectroscopy, mass spectrometry, and evaluation of its photochemical properties. In the absorption spectra, there were two characteristic bands for porphyrazine molecules—the Soret (short wavelengths) and the Q-bands (long wavelengths). The obtained porphyrazine showed a strong Soret band between 350 and 400 nm and a similar intense Q band in the range 650–700 nm. Pz **4** had a low ability to generate singlet oxygen, with Φ_Δ_ values of 0.064 and 0.004 in DMF and DMSO, respectively, as measured with an indirect method using DPBF. No emission properties were recorded for the porphyrazine. The electrochemical measurements indicated that ferrocene-based redox processes of compounds **1**, **2**, and **4** were shifted towards more positive potentials in comparison to unsubstituted Fc. This positive shift can be assigned to the electron-withdrawing effect of the oxohexyl groups. The most positive peak potential observed for **4** (0.34 V vs. Fc^+^/Fc) was probably correlated to the strongest inductive effect of the eight substituents existing close together in the **4** macrocycle. Adsorption phenomena existed at carbon-based electrodes in the case of **4**. Such a behavior opens up further possibilities for applying **4** in the development of modified electrodes with electroactive redox Fc^+^/Fc couples.

## Figures and Tables

**Table 1:** UV-Vis absorption maxima (λ_Abs_) with logarithms of molar absorption coefficients (log ε) and quantum yields of the singlet oxygen generation (Φ_Δ_) for Pz **4**.

Solvent	λ_1Abs_ (log ε)	λ_2Abs_ (log ε)	Φ_Δ_
Dichloromethane	378 (4.48)	675 (4.86)	-
*N*,*N*-Dimethylformamide	380 (4.84)	671 (4.86)	0.064
Dimethyl sulfoxide	380 (4.84)	674 (4.88)	0.004

## Data Availability

Not applicable.

## Supplementary Materials

The following supporting information can be downloaded at https://www.mdpi.com/article/10.3390/molecules28135215/s1: Figure S1: ^1^H and (^13^C) chemical shift values [ppm] of **3** and key correlations observed in NMR spectra; Figure S2: ^13^C NMR spectrum of **3** (201 MHz, DMSO-*d*_6_, 298 K); Figure S3: ^1^H and (^13^C) chemical shift values [ppm] of **4** and key correlations observed in NMR spectra; Figure S4: ^13^C NMR spectrum of **4** (201 MHz, DMSO-*d*_6_, 298 K); Table S1: ^1^H and ^13^C NMR data obtained for **3**, including key correlations determined from ^1^H-^13^C HSQC and ^1^H-^13^C HMBC spectra; Table S2: ^1^H and ^13^C NMR data obtained for **4**, including key correlations determined from ^1^H-^13^C HSQC and ^1^H-^13^C HMBC spectra.
