# Supplementary material for: Synthesis, Electrochemical and Photochemical Properties of Sulfanyl Porphyrazine with Ferrocenyl Substituents
Source: Molecules. 2023 Jul 5;28(13):5215. doi: 10.3390/molecules28135215 (PMC10343546; doi:10.3390/molecules28135215)
Supplement: Supplementary file 1 [file molecules-28-05215-s001.zip › molecules-2477178-supplementary.pdf]

Supplementary data

for

Synthesis, electrochemical and photochemical properties of sulfanyl porphyrazine with ferrocenyl substituents

Mina Hassani [1], Amanda Leda [2], Weronika Porolnik [3], Michal Falkowski [1,*], Tomasz Rebis [2,*], Jaroslaw Piskorz [3], Lukasz Popenda [4], Michał Wicinski [5], Dariusz T. Mlynarczyk [6], Nejat Düzgüneş [7], Michal P. Marszall [1]

[1] Department of Medicinal Chemistry, Collegium Medicum in Bydgoszcz, Faculty of Pharmacy, Nicolaus Copernicus University in Torun, Dr. A. Jurasza 2, 85-089 Bydgoszcz, Poland; 503341@doktorant.umk.pl (M.H.); mmars@cm.umk.pl (M.P.M.)

[2] Institute of Chemistry and Technical Electrochemistry, Poznan University of Technology, Berdychowo 4, 60-965 Poznan, Poland; amanda.leda@doctorate.put.poznan.pl (A.L.)

[3] Chair and Department of Inorganic and Analytical Chemistry, Poznan University of Medical Sciences, Rokietnicka 3, 60-806 Poznan, Poland; w.porolnik@op.pl (W.P.); piskorzj@ump.edu.pl (J.P.)

[4] NanoBioMedical Centre, Adam Mickiewicz University, Wszechnicy Piastowskiej 3, 61-614 Poznan, Poland; lpopenda@amu.edu.pl

[5] Department of Pharmacology and Therapy, Collegium Medicum in Bydgoszcz, Faculty of Medicine, Nicolaus Copernicus University in Torun, Curie Sklodowskiej 9, 85-094 Bydgoszcz, Poland; (MW) michal.wicinski@cm.umk.pl

[6] Chair and Department of Chemical Technology of Drugs, Poznan University of Medical Sciences, Grunwaldzka 6, 60-780 Poznan, Poland; mlynarczykd@ump.edu.pl (DTM)

[7] Department of Biomedical Sciences, Arthur A. Dugoni School of Dentistry, University of the Pacific, San Francisco, CA 94103, USA

CORRESPONDING AUTHORS*: m.falkowski@cm.umk.pl (Michal Falkowski), tomasz.rebis@put.poznan.pl (Tomasz Rebis)

Table of Contents

1. HPLC purity of porphyrazine (4)....................3

2. NMR study....................8

Figure S1. ^1H and (^{13}C) chemical shift values [ppm] of **3** and key correlations observed in NMR spectra.
Table S1. ^1H and ^{13}C NMR data obtained for **3** including key correlations determined from ^1H-^{13}C HSQC and ^1H-^{13}C HMBC spectra.
Figure S2. ^{13}C NMR spectrum of **3** (DMSO-d_6, 298 K).
Figure S3. ^1H and (^{13}C) chemical shift values [ppm] of **4** and key correlations observed in NMR spectra.
Table S2. ^1H and ^{13}C NMR data obtained for **4** including key correlations determined from ^1H-^{13}C HSQC and ^1H-^{13}C HMBC spectra.
Figure S4. ^{13}C NMR spectrum of **4** (Pyridine-d_5, 298 K).

1. HPLC purity

The purity of macrocycle **4** was determined by HPLC analysis using an Agilent 1200 instrument equipped with a UV-DAD detector. The chromatographic separation was obtained on an octadecylsilane-coated column, 150 mm × 4.6 mm, 5 μm (Eclipse XDB-C18, Agilent), using gradient elution conditions at a flow rate of 1.0 mL/min. Band dispersion, and additional peaks from aggregates significantly hampered HPLC analysis. The best conditions for the compound are shown below. Peaks of minor components were detected, but the impurity content never exceeded 5% of the total signal intensity.

Porphyrazine 4

Configuration 1

	phase		
time [min]	methanol	tetrahydrofuran	water
0	80	10	10
3	80	10	10
4	50	50	0
15	50	50	0

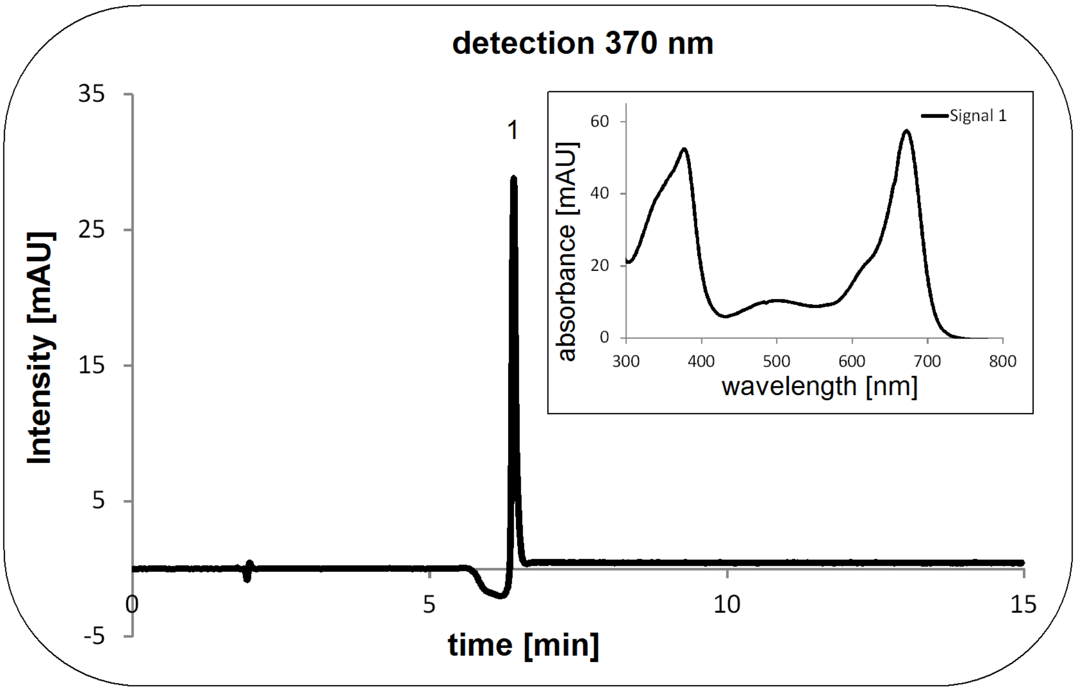

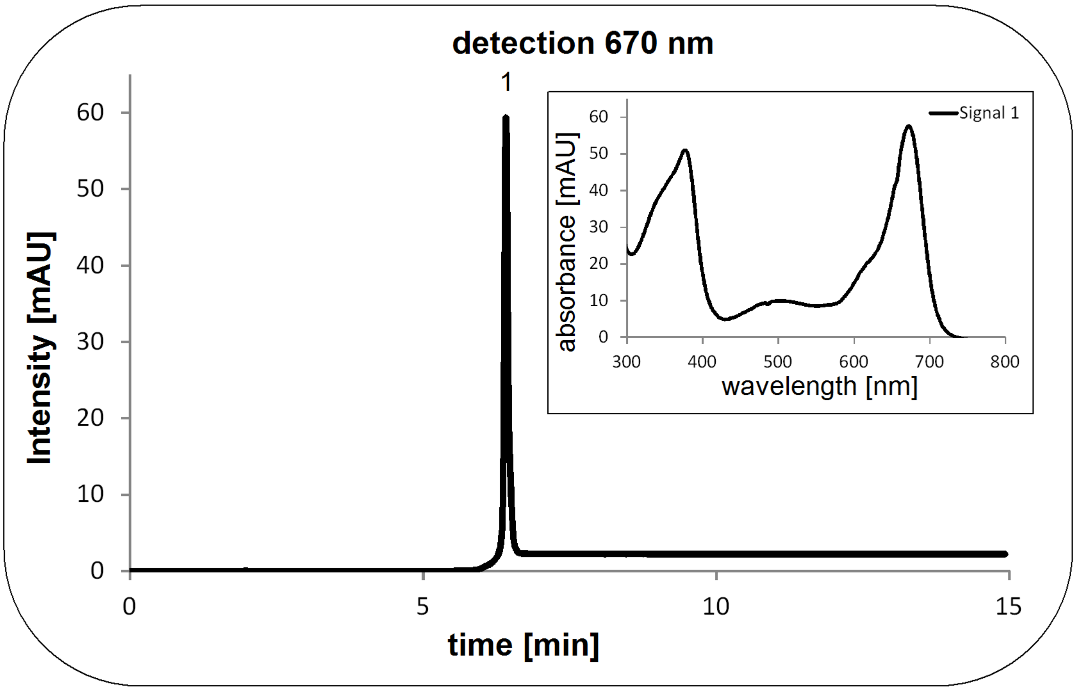

results			
signal	retention time [min]	area	purity [%]
detection 370 nm			
1	6.4	147.5	100.0
detection 670 nm			
1	6.4	288.1	100.0

Configuration 2

	phase			
time [min]	acetonitrile	methanol	water	tetrahydrofuran
0	35	35	15	15
3	35	35	15	15
4	15	15	0	70
15	15	15	0	70

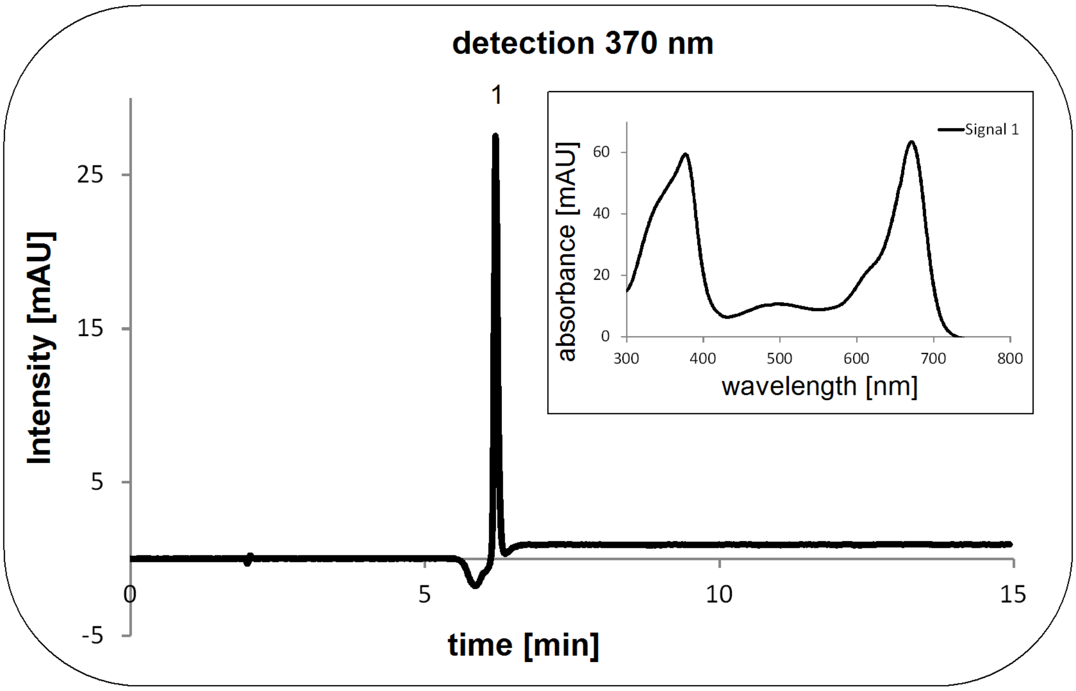

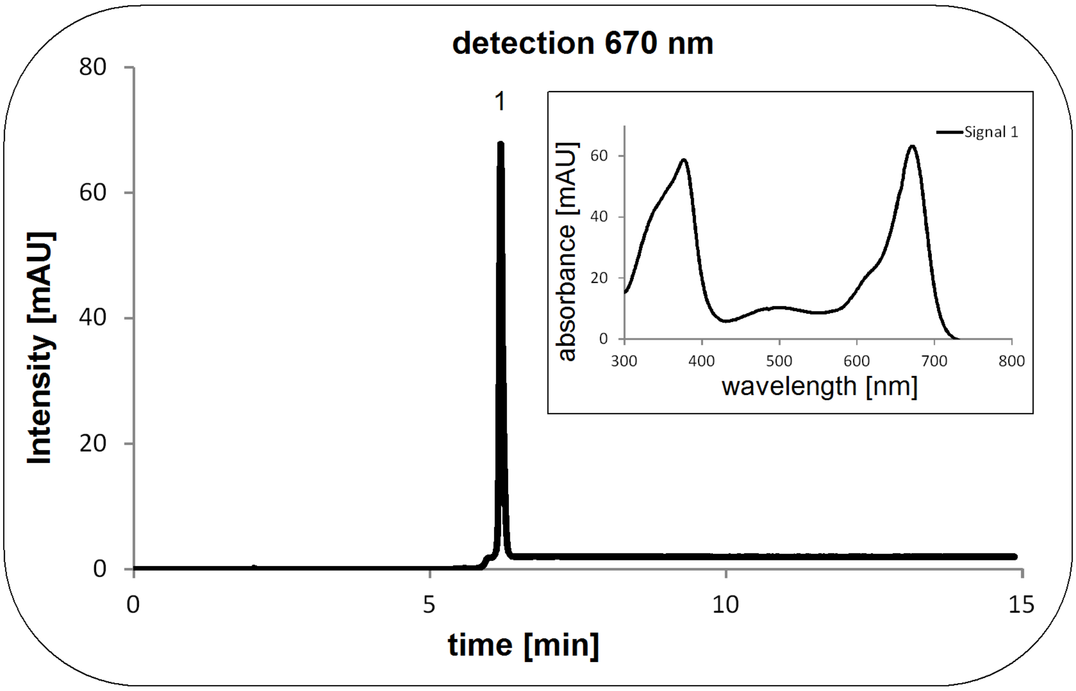

results			
signal	retention time [min]	area	purity [%]
detection 370 nm			
1	6.2	127.4	100.0
detection 670 nm			
1	6.2	300.7	100.0

Configuration 3

phase			
time [min]	methanol	dichloromethane	water
0	90	5	5
3	90	5	5
4	10	90	0
15	10	90	0

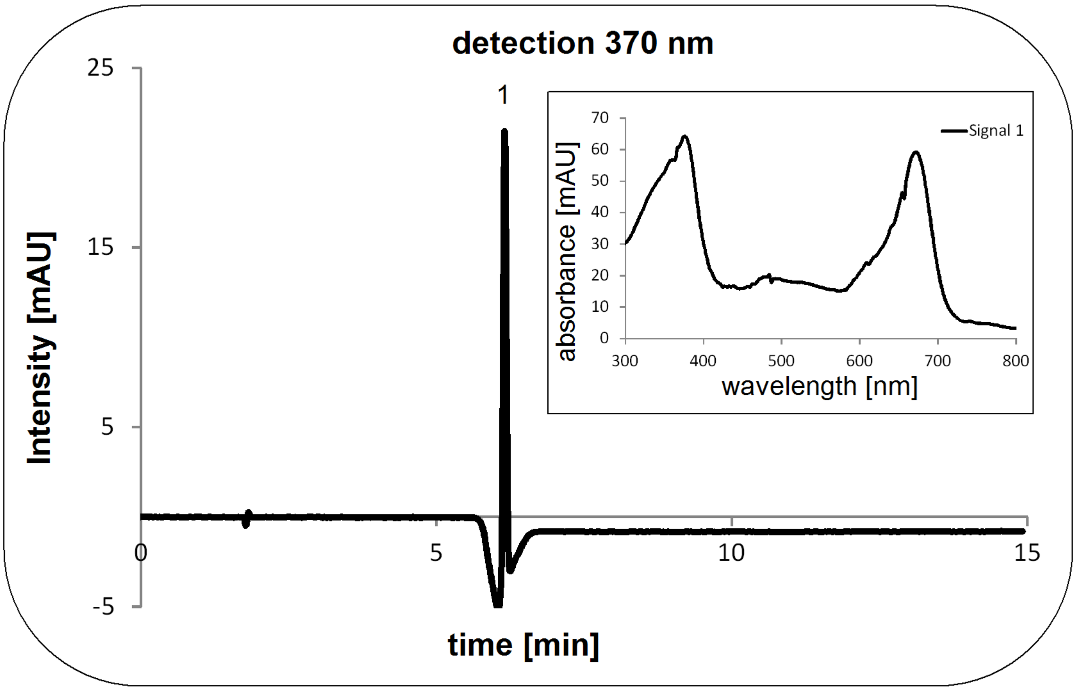

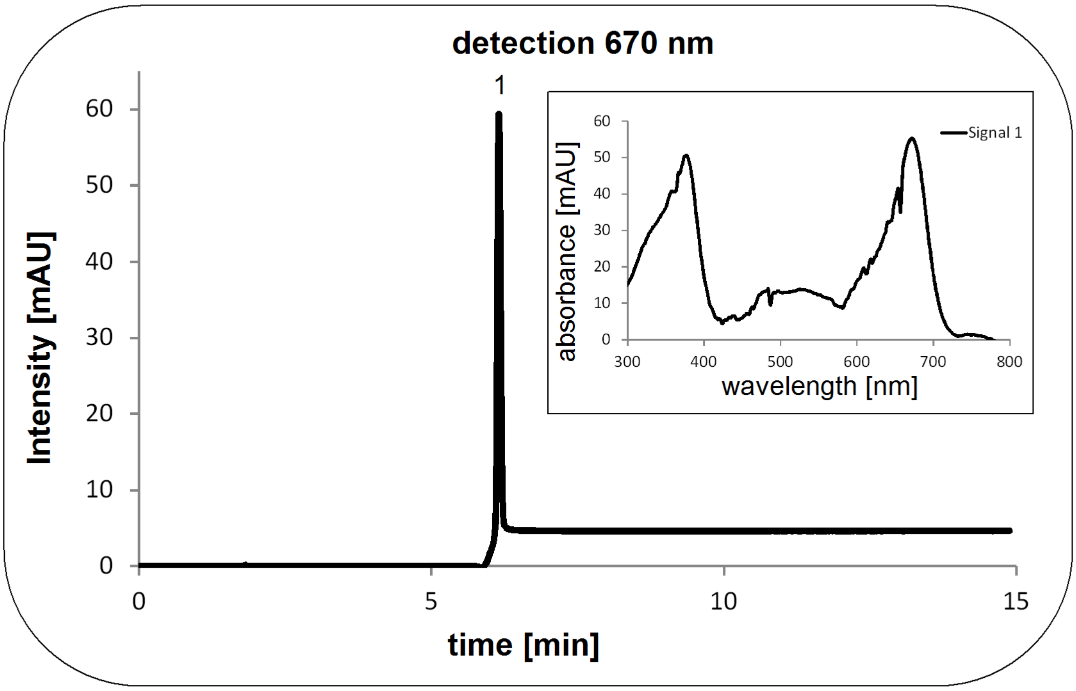

results			
signal	retention time [min]	area	purity [%]
detection 370 nm			
1	6.2	105.0	100.0
detection 670 nm			
1	6.2	246.4	100.0

2. NMR study

2.1. NMR data of 3

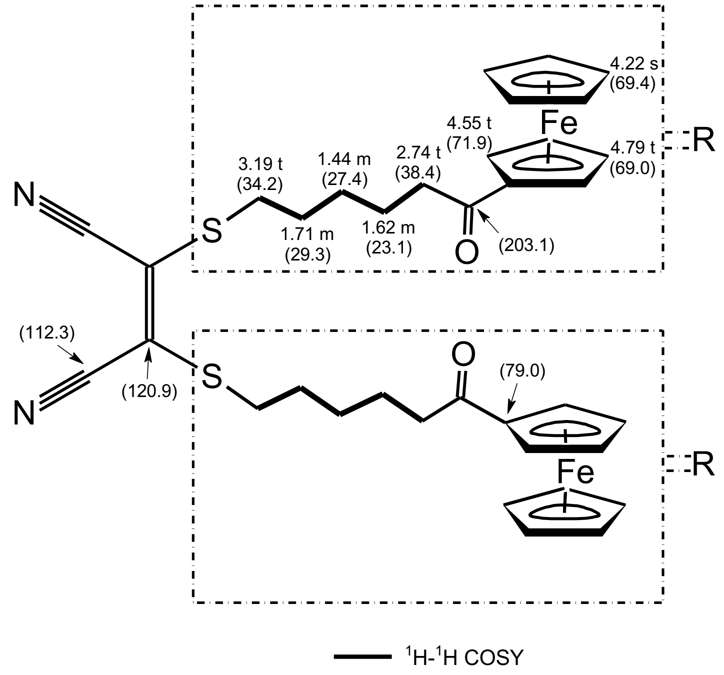

Figure S1. NMR data of **3**: ^{1}H and (^{13}C) chemical shift values [ppm] and key correlations observed in NMR spectra. Bold lines: ^{1}H-^{1}H COSY; Dashed arrows: ^{1}H-^{13}C HMBC.

Table S1. ^{1}H and ^{13}C NMR data obtained for **3** including key correlations determined from ^{1}H-^{13}C HSQC and ^{1}H-^{13}C HMBC spectra.

**δ_H (ppm)**	**Multiplicity (J_{H-H} in Hz)**	**^{1}H-^{13}C HSQC δ_C (ppm)**	**^{1}H-^{13}C HMBC δ_C (ppm)**		
4.79	t (1.8)	69.0	69.0	71.9	79.0
4.55	t (1.8)	71.9	69.0	71.9	79.0
4.22	s	69.4	69.4		
3.19	t (7.3)	34.2	27.4	29.3	120.9
2.74	t (7.1)	38.4	23.1	27.4	79.0
			203.1		
1.71	m	29.3	23.1	27.4	34.2
1.62	m	23.1	27.4	29.3	38.4
			203.1		
1.44	m	27.4	23.1	29.3	34.2
			38.4		
Other quaternary carbon atoms: 120.9, 112.3 (ppm)					

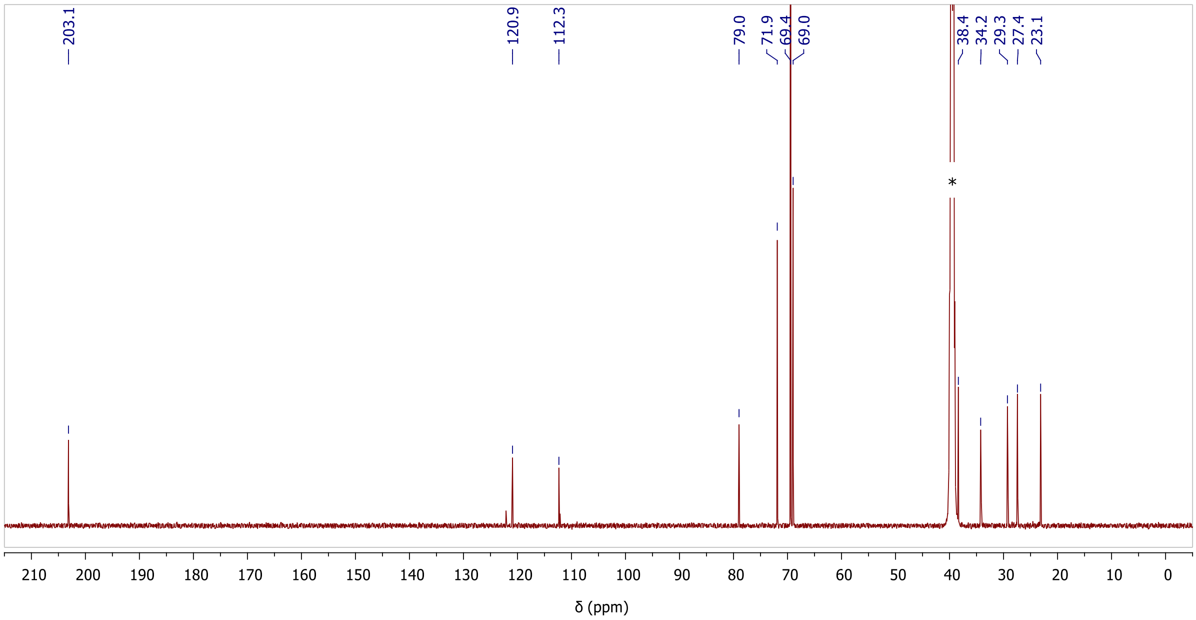

Figure S2. ^{13}C NMR spectrum recorded for **3** (DMSO-d_6, 298 K). The symbol * indicates DMSO-d_6 peak.

2.2. NMR data of 4

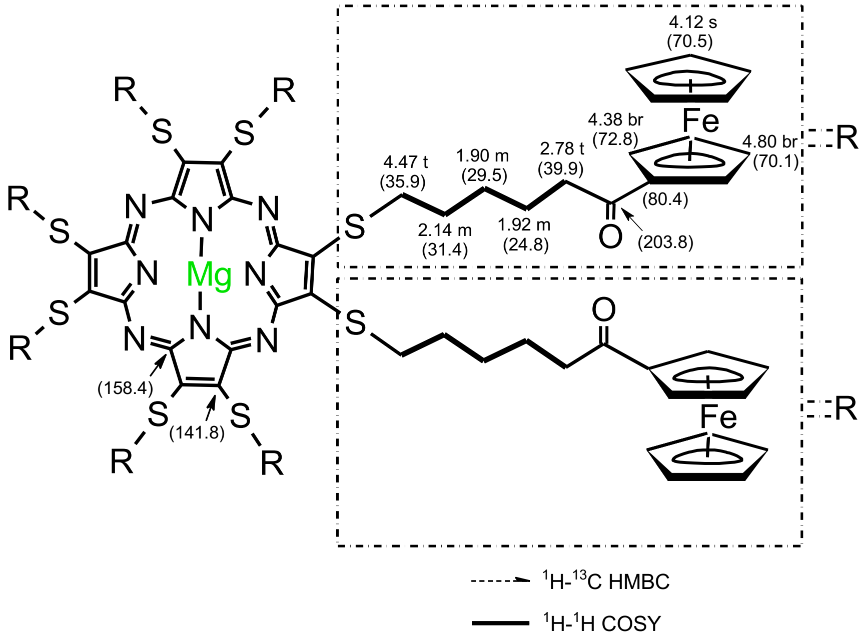

Figure S3. NMR data of **4**: ^{1}H and (^{13}C) chemical shift values [ppm] and key correlations observed in NMR spectra. Bold lines: ^{1}H-^{1}H COSY; dashed arrows: ^{1}H-^{13}C HMBC.

Table S2. ^{1}H and ^{13}C NMR data obtained for **4** including key correlations determined from ^{1}H-^{13}C HSQC and ^{1}H-^{13}C HMBC spectra.

δ_H (ppm)	Multiplicity ($J_{H\text{-}H}$ in Hz)	^{1}H-^{13}C HSQC δ_C (ppm)	^{1}H-^{13}C HMBC δ_C (ppm)		
4.80	br	70.1	70.1	72.8	80.4
4.47	t (7.0)	35.9	29.5	31.4	141.8
4.38	br	72.8	70.1	72.8	80.4
4.12	s	70.5	70.5		
2.78	t (6.1)	39.9	24.8	29.5	203.8
2.14	m	31.4	24.8	29.5	35.9
1.92	m	24.8	29.5	31.4	39.9
			203.8		
1.90	m	29.5	24.8		
Other quaternary carbon atoms: 158.4, 141.8 (ppm)					

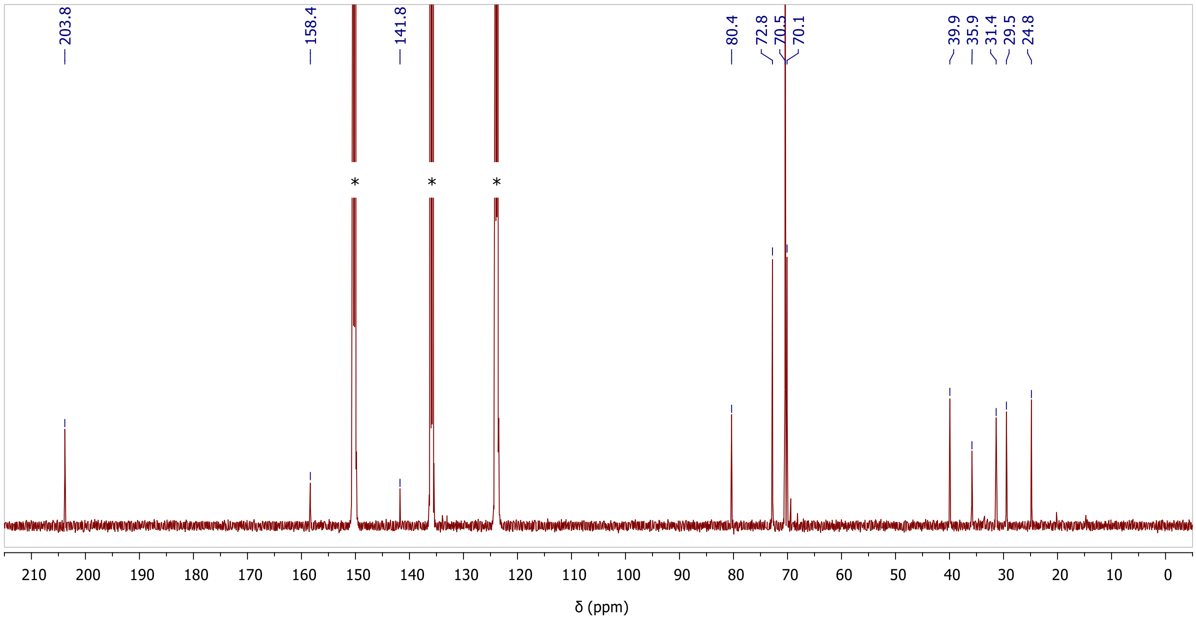

Figure S4. ^{13}C NMR spectrum recorded for **4** (pyridine-d_5, 298 K). The symbol * indicates pyridine-d_5 peaks.
